# Bacteriuria and urinary schistosomiasis in primary school children in rural communities in Enugu State, Nigeria, 2012

**DOI:** 10.11694/pamj.supp.2014.18.1.4169

**Published:** 2014-07-21

**Authors:** Okechukwu Paulinus Ossai, Raymond Dankoli, Chimezie Nwodo, Dahiru Tukur, Peter Nsubuga, Daniel Ogbuabor, Osaeloka Ekwueme, Godwin Abonyi, Echezona Ezeanolue, Patrick Nguku, Douglas Nwagbo, Suleiman Idris, George Eze

**Affiliations:** 1 Nigeria Field Epidemiology and Laboratory Training Programme (NFELTP), Abuja, Nigeria; 2 Department of Community Medicine, Ahmadu Bello University, Zaria, Nigeria; 3 Global Public Health Solutions, Atlanta, USA; 4 Department of Community Medicine, University of Nigeria, Nsukka, Nigeria; 5 Department of Paediatrics, University of Nevada School of Medicine, USA; 6 Ministry of Health, Enugu State, Southeast, Nigeria

**Keywords:** Bacteriuria, co-infection, urinary schistosomiasis, Enugu State

## Abstract

**Introduction:**

According to a study conducted in1989, Enugu State has an estimated urinary schistosomiasis prevalence of 79%. Recently, studies have implicated bacteriuria co-infection in bladder cancer. These bacteria accelerate the multi-stage process of bladder carcinogenesis. Knowledge about the prevalence of this co-infection is not available in Enugu and the information provided by the 1989 study is too old to be used for current decision making.

**Methods:**

We carried out a cross-sectional survey of primary school children aged5-15years, who were randomly selected through a multi stage sampling method using guidelines recommended by WHO for schistosomiasis surveys. An interviewer administered questionnaire was used to collect data on demography, socioeconomic variables and clinical presentations. Urine samples were collected between 10.00am and 2.00pm. Each sample was divided into two: (A) for prevalence and intensity using syringe filtration technique and (B) for culture. Intensity was categorized as heavy (>50ova/10mls urine) and light (<50ova/10mls urine). Significant bacteriuria was bacteria count ≥ 105 colony forming units/ml of urine.

**Results:**

Of the 842 pupils, 50.6% were females. The prevalence of urinary schistosomiasis was 34.1%. Infection rate was higher(52.8%) among 13-15 years(Prevalence Ratio = 2.45, 95% Confidence Interval 1.63-3.69). Heavy infections wad 62.7% and egg count/10mls urine ranged from 21-1138. Significant bacteriuria among pupils with urinary schistosomiasis was 53.7% compared to 3.6% in the uninfected(PR = 30.8,95% CI 18.91- 52.09). The commonest implicated organism was Escherchia coli.

**Conclusion:**

We found high prevalence of bacteriuria co-infection among children with urinary schistosomiasis in Enugu State. This underscores the need for concurrent antibiotics administration and follow-up to avert later complications.

## Introduction

Schistosomiasis is a neglected parasitic tropical disease caused by a trematode of the genus schistosoma and it is one of the major public health problems facing developing countries. School-aged children are at the greatest risk of acquiring schistosomiasis and boys are more affected than girls [1]. The disease affects both the gastro-intestinal tract through infection by Schistosoma mansoni, japonicum, intercalatum or mekongi, causing bloody stool and ascites, and the urinary tract through infection by Schistosoma haematobium which causes haematuria. Schistosomiasis is mostly found in Asia, Africa, and South America in areas where the water contains fresh-water snails such as Biomphalaria and Bulinus which carry the parasite. Globally, over 600million people are at risk with over 200 million infections in 76 countries annually. More than 20 million people come down with the disease following infection and it is estimated that about 20,000 deaths are attributed to the disease annually [2]. Africa accounts for over 85% of schistosomiasis burden. Nigeria is the most endemic country in the world for urinary schistosomiasis with an estimated 25.83million people infected [3, 4]. In Enugu state which is located in south eastern Nigeria, haematuria is a common complaint in many communities. A study in a focal community in 1989 revealed a prevalence of urinary schistosomiasis of 79% [5]. Several studies have implicated bacteriuria co-infection with urinary schistosomiasis in the aetiology of bladder cancer and other complications [6]. Studies have shown that it may take up to 10-20 years after initial co- infection for terminal complications such as renal failure and squamous cell carcinoma of the bladder to develop [7, 8]. The potential association of the urinary schistosomiasis with other infectious diseases (e.g., urinary bacteria) is so far not well understood. Control measures that are instituted by various public health agencies pay little attention to the complexity of schistosomiasis morbidity and its assumed dependency on co-infection with bacteriuria [9, 10]. When the mucosal barrier is broken down which happens with urinary schistosomiasis, the urinary tract becomes an easy target for invading bacteria. These bacteria accelerate the multi-stage process of bladder carcinogenesis as experimental evidence has shown by the formation of N-nitroso compounds, produced from amine precursors and nitrate in urine during bacterial infections [11, 12]. Some of the compounds like N-butyl-N-(4-hydroxy butyl) nitrosamine (BHBN) and N-methyl-N-nitro-urea (NMU) are known bladder carcinogens [12]. Systematic knowledge about bacterial co-infection and schistosomiasis in the 5-15years age group is scanty which is understandable since methods for schistosomiasis surveys are not optimal for detecting bacteriuria [13]. Against this background, current information on the prevalence of urinary schistosomiasis, as well as associated bacteriuria co-infection is necessary for effective control of the disease and in preventing later complications. We therefore investigated the current prevalence of urinary schistosomiasis and co-infection with bacteriuria in Enugu in order to provide information for public health action.

## Methods

**Study area:** We conducted the study in three randomly selected rural Local Government Areas (LGA) of Enugu state, south- eastern part of Nigeria. The selected LGAs were Aninri, Nkanu east, and Uzouwani with a population of 236,221, 253,591and 227,150 respectively projected from 2006 Nigerian census figures [14]. These LGAs also had 66, 70, and 69 public primary schools respectively.

**Study Population:**: We recruited primary school pupils aged 5-15 years for this study.

**Study Design/Sample Size Estimation:** We conducted a cross-sectional survey. We adopted the WHO guideline for survey of schistosomiasis and other soil transmitted infections. The guideline recommends that when a survey is organized to assess the need for control measures (e.g., prevalence and intensity), a sample size of 200- 250 people is adequate [15]. However, when a study is to evaluate parameters other than prevalence and intensity (e.g., the relationship of schistosomiasis with other diseases), a larger sample size is required. We chose a sample size of 300 pupils from each LGA, giving a pooled sample size of 900 pupils for the three selected LGAs in the study.

**Inclusion/Exclusion Criteria:** Primary school Pupils below the age of 5 years and above 15 years were excluded from the study.

**Sampling Method:** A multi- stage sampling method was used. From each senatorial zone, an LGA was randomly selected, among the rural LGAs in the area. Three primary schools were randomly selected from the list of public primary schools in each of the LGAs. The education policy in Enugu state is that the maximum number of pupils in a class is 30 pupils. We targeted 100 pupils per school and we randomly selected four classes in each school. All the pupils who met the inclusion criteria (and consented or for whom we had consent from their parents) were enrolled in each selected class.

**Data Collection:** Disease Surveillance and Notification Officers (DSNO's) and focal persons were trained and used as interviewers. Information on socio-economic, demographic, risk factors and clinical presentations was collected from every participant in the study using standardized, pretested questionnaires translated into a local dialect.

### Laboratory methods

**Sample Collection:** A 20 millilitre clean catch, mid-stream urine sample was collected in 50mls capacity autoclaved wide mouthed leak-proof universal containers by subjects themselves, who were previously carefully instructed with illustration aids. Samples were collected between 10.00am and 2.00pm which is the peak period for shedding of schistosoma eggs. Each sample collected was inspected for visible haematuria and turbidity. The samples were appropriately labeled with identification numbers and placed in a cold box with ice packs, immediately after collection. Laboratory analyses were carried out at Enugu State University of Science and Technology (ESUT) Teaching Hospital, Parklane, Enugu.

**Laboratory Procedures:** Each sample was divided into two fractions A and B of 10mls each. Fraction A was investigated for the presence of Schistosoma haematobium ova. It was first subjected to a commercially prepared reagent strip-Multistix 8 SG reagent strip (Siemens Lot 04200746) to check for the specific gravity, haematuria, leucocyturia, and proteinuria. The strip was dipped into each urine sample and the colour change was matched with standard colours by the side of the container of the reagent strips. Urine specific gravity values of 1.000 to 1.020 were taken as normal while values> 1.020 were considered significant for a disease condition [16]. Thereafter the urine fraction was filtered through a 13-mm-diameter polycarbonate membrane with a 20 micro-millimetre pore size. The filter was removed with forceps, placed on a glass slide and stained with a drop of 50% lugols iodine and examined under x40 light microscope and the number of eggs of S. haematobium eggs were counted and expressed as eggs / 10 ml of urine. Intensity was reported as the number of ova per 10mls of urine and categorized as “light” when < 50ova/10mls were found and “heavy” when >50ova/10mls of urine were found. Fraction B was cultured on blood agar and cystine-lactose-electrolyte-deficient (CLED) agar plates respectively using the standard methods [17]. All bacterial isolates thus obtained were characterized by using the standard methods [18]. Significant bacteriuria was described as bacteria count of equal or greater than 105 colony forming units per ml of urine (cfu/ml).

**Quality Control:** Quality control was undertaken to verify the consistency of the microscopic readings according to WHO guidelines. Before the survey, a day was spent evaluating the consistency of egg counting among laboratory technicians by preparing 10 slides and comparing the reading of each slide by each laboratory technician with that of the team leader. A discrepancy of up to 10% for egg counts was taken as normal, but if the discrepancy was larger, the reasons were identified and corrected. When one of the microscopists presented readings which were consistently different to those of the others in the team, he or she was excluded from the team. Each day during the survey, the team leader read 10% of the slides of each microscopist without prior knowledge of the result. In the case of a discrepancy larger than 10%, the slide was discussed by the two readers, and further slides examined to avoid repeated errors.

**Data Analysis:** Microsoft Excel was used for data analysis to calculate frequencies. Categorical variables were compared with the Chi-square test and odds ratios using Epi-info version 3.5.1

**Ethical Consideration:** Approval for this study was obtained from the Ethical Review Committee of Postgraduate Institute of Medical Research and Training of the University of Nigeria Teaching Hospital, Enugu State. We also obtained approval from the health and education departments of the three respective LGAs where the study was carried out. Oral informed consent was obtained from parents through the Parents Teachers Associations of the respective schools. Participation by pupils was voluntary after obtaining assent. Information collected from participants was maintained with utmost confidentiality as names were not used on any sample but codes.

## Results

A total of 842 primary school children out of a target of 900 participated in the study from the 3 LGAs selected giving a response rate of 93.6%. They were 262,309 and 271 pupils from Aninri, Nkanu east, and Uzouwani respectively. All were above the WHO recommendation of 200-250 for prevalence studies. The majority (51.8%) of the respondents were 9-12 years. The ratio of male respondent to female was almost 1(416/426) (Table 1). We found a pooled prevalence of urinary schistosomiasis of 34.1%. The highest prevalence of urinary schistosomiasis (52.8%) was among 13-15year old age-group (Table 2). The infection rate was higher in males (37%) than females (31.2%) but not statistically significant(p = 0.08). The infection rate was higher in primary schools in Nkanu east (43.0%) than the other two LGAs. The highest prevalence of 66% was among respondents from one of the schools in Nkanu east LGA while the lowest (7.6%) was in a school in Uzouwani LGA. Intensity ranged from 21 to 1138eggs/10mls of urine. The mean egg count was 348/10mls urine (Standard Deviation 178.1). Heavy intensity (eggs > 50/10mls of urine) accounted for over 62% of cases. The percentage heavy intensity was higher in females than males. Among the different age groups, 9-12years age group had the highest number of pupils with heavy intensity (Table 3). The overall prevalence of significant bacteriuria was 53.7% among those infected with urinary schistosomiasis and 3.6% in those that were not infected with urinary schistosomiasis. The presence of ova in urine was found to have significant association with bacteriuria among the study group (p value < 0.001). There was no difference between prevalence of significant bacteriuria among male and females with urinary schistosomiasis (Table 4) The predominant urinary pathogens associated with urinary schistosomiasis were Escherichia coli, Staphylococcus aureus, and Klebsiella species and there were no gender differences in the types of urinary bacteria pathogens.

**Table 1:** Age group and gender distribution of respondents among Primary School Children in Aninri, Nkanu East and Uzouwani LGAs of Enugu State, Nigeria, 2012

Age group	Male (N/%)	Female (N/%)	Total (N/%)
5-8years	162(38.9)	136(31.9)	298(35.4)
9-12years	190(45.7)	246(57.8)	436(51.8)
13-15years	64(15.4)	44(10.3)	108(12.8)
Total	416(100)	426(100)	842(100)

**Table 2:** Prevalence of Urinary Schistosomiasis by Age Group and Gender among Primary School Children in Aninri, Nkanu East and Uzouwani LGAs of Enugu State, Nigeria, 2012

Age-group	Male	Female	Total	P-value
	No (%) Infected	No (%) Infected	No (%) Infected	
5-8 years	43(26.5)	29(21.3)	72(24.2)	0.30
9-12 years	71(37.4)	87(35.4)	158(36.2)	0.67
13-15 years	40(62.5)	17(38.6)	57(52.8)	0.02*
Total	154(37.0)	133(31.2)	287(34.1)	0.08

* Significant

**Table 3:** Classification of Infection by Intensity in Different Age Groups among Primary School Children in Aninri, Nkanu East and Uzouwani LGAs of Enugu State, Nigeria, 2012

Parameter	Male		Female		Total	
Age Group	Light infect	Heavy infect	Light infect	Heavy infect	Light infect	Heavy infect
5-8 years	31	12	8	21	39	33
9-12years	26	45	23	64	49	109
13-15years	15	25	4	13	19	38
**Total**	72	82	35	98	107(37.3%)	180(62.7%)

**Table 4:** Prevalence of Bacteriuria among Primary School Children Infected with Urinary Schistosomiasis by Gender, in Aninri, Nkanu East and Uzouwani LGAs in Enugu State, Nigeria, 2012

LGA	Male N = 416				Female N = 426			
	Ova present	Ova present with bact/%	Ova absent with bact	p-value	Ova present	Ova present with bact/%	Ova absent with bact	p-value
Aninri	44	29(65.9)	5	<0.001	51	16(31.4)	4	<0.001
Nkanu east	73	36(49.3)	2	<0.001	60	39(65)	4	<0.001
Uzouwani	37	19(51.4)	2	<0.001	22	15(68.2)	2	<0.001
**Total**	154	84(54.6)	9	<0.001	133	70(52.6)	11	<0.001

## Discussion

We found that of the 287 pupils with urinary schistosomiasis in our study population, 53.7% had bacteriuria while among those not infected with urinary schistosomiasis only3.6% had bacteriuria. The presence of Schistosoma haematobium ova was significantly associated with high prevalence of bacteriuria (p value< 0.001). This result is comparable to the findings in 2008 in Ngbo west - a similar rural community in the same socio-cultural and ecological zone with known high prevalence of urinary schistosomiasis where a bacteriuria prevalence of 48.3% was reported among people with urinary schistosomiasis by C.J. Uneke et al [19]. The different bacteriuria prevalence levels among schistosomiasis infected and uninfected school children is also similar to findings in a survey of school children(5-16years) in Egypt in1978 by Laughlin et al [20] where they found that the prevalence of bacteriuria was 10 times higher in areas endemic for urinary schistosomiasis than in non-endemic areas. This high rate of concomitant bacteriuria in children where ordinarily significant bacteriuria is known to be low could be attributed to urinary schistosomiasis. During infestation, as Schistosoma haematobium eggs are being ejected into urine, some blood also leaves with the eggs concurrently from the bladder. Blood being a potential culturing medium can encourage the flourishing of bacteria organisms in the urinary tract. This co-infection has been documented as potential risk factor in the incidence of squamous cancer of the bladder in later years [7, 8, 21–23]. Our study did not show any significant difference in the prevalence between the males and females. Significant bacteriuria is known to have no sex preference at younger age group. The most common organisms isolated in the study were Escherichia coli, Klebsiella and Staphylococcus auerus -all are nitrate reducing bacteria. This is similar to the findings in some other states in Nigeria such as study by Uneke et al in Ebonyi State [19] and Normosi et al in Ogben rural community of Edo state [24].

Our study also showed a pooled prevalence of 34.1% of urinary schistosomiasis among the school age children in the three randomly selected LGAs. This places the area in category 2 (20-50% prevalence) and qualifies them for targeted mass drug administration according to WHO guidelines. WHO recommends universal treatment for communities in category 1 (prevalence> 50%). In this case the whole community is treated irrespective of age, sex, infection status, or other social characteristics, and treatment campaigns must be conducted once a year. For communities in category 2 (prevalence 20-50%), targeted treatment is recommended. Here the groups identified for treatment are school-age children, and the treatment should be organized every 1-2 years. Unfortunately, this guideline is not being followed in the state as the last mass drug administration was in 1998. The 34.1% prevalence is in contrast with 79% prevalence obtained by Ozumba et al in Amagunze, Nkanu east LGA of the state in 1989 [5] and 79.4% by Uneje et al in nearby Ezza North LGA of Ebonyi state lying in the same geographical zone of Nigeria [19]. The differences in prevalence among these studies could be attributed to reduction in water contact practices due to availability of some boreholes in the areas as well as residual awareness created during the period of mass drug administration. The differences could also be attributed to different diagnostic techniques used; the sedimentation methods were used in the two referenced studies while syringe filtration technique was used in our study. When the prevalence was stratified according to LGAs, Nkanu east had the highest prevalence of 43% compared to 36% and 21% for Aninri and Uzouwani LGAs respectively. It was also observed that the school that recorded the highest prevalence was also in Nkanu east LGA. This is not surprising as the previous studies by Ozumba et al in 1989 had shown that the disease is endemic around that area [5]. However, all the figures obtained for prevalence in all the LGAs sampled were above the Nigerian national mean of 13% according to findings of Ofoezie I.E in 2002 [25]. There was no significant difference in the prevalence of urinary schistosomiasis between males (37.0%) and females (31.2%). This may be an indication that both gender and all the age groups were being equally exposed to infection through water contacts as there may be no cultural barrier against any gender or group. The fact that both genders had similar prevalence agrees with previous reports that Schistosoma haematobium infection is not gender-specific in many parts of Nigeria [26, 27]. There was significant difference in the infection rate among the age groups with 13-15years recording the highest prevalence of 52.8%. This is similar to findings in other studies elsewhere e.g., U.S. Ugbomoiko et al in a study in two peri-urban communities in south western Nigeria [28]. This could be explained by the fact that children are more adventurous around this age and therefore more likely to have more contact with water bodies.

Our findings were different from the findings of Morenikei O.A. et al in Ogun State southwestern Nigeria in 2011 where the peak of infection was at 8-10 years [29]. Our study showed that in Enugu State, the risk of schistosomiasis infection is wide spread as evidenced by the prevalence in all the surveyed LGAs. In our study, heavy intensity accounted for 62.7% of positive cases of urinary schistosomiasis. This is comparable to 74.4% obtained in Ikpeshi, Akoko Edo LGA and 59.5% in Ogben, Owan East LGA both of Edo State by Normosi et al in 2001 and 2007 respectively [30, 31]. Our findings differ from the findings in a study among school children by Atupelep et al in a similar rural community in Malawi where heavy intensity was found to be 2.4% [32]. This might be attributed to differences in ecological zones and probably the effectiveness of the intervention programmes in Malawi. We found that more females than males had heavy intensity (p value < 0.001), in Enugu State. Young girls do most of the domestic activities which include fetching water for general domestic uses and going to streams for laundry and they therefore are more exposed to water bodies than their male counterparts. The percentage heavy intensity was also significantly observed to be higher among the 9-12 year age group. The higher rate of heavy intensity at this age group may be due to high water contact activities in the phase of low immunity. Immunity to diseases is believed to develop with age and exposure. At this age, the immune system of children have not fully developed and they also have increasing adventurous tendencies leading them to frequent water contacts and hence more exposure with low immunity. Children above 13 years accounted for 12.8% of the respondents in our study. The high number of children in this age bracket who were still in primary school points to delayed schooling which probably could be attributed to schistosomiasis infection [33]. The good response rate also gives credence to the general belief that children give better compliance and that the community sees urinary schistosomiasis as a problem that needs solution [34, 35].

Generalization of our results is limited by the fact that only children who were enrolled in schools were studied. School age children who engage in other activities such as farming or fishing who are even more at risk were not studied. It is likely that they may even have higher prevalence of urinary schistosomiasis and bacteriuria. We also did not check for other possible factors that predispose children to bacteriuria in rural communities such as poor sanitary habits. Furthermore, we did not establish the incidence of bladder cancer in Enugu State, which could lend credence to our study's main hypothesis.

## Conclusion

We concluded that there was high prevalence of bacteriuria co-infection among school age children with urinary schistosomiasis in Enugu state, Nigeria. This co-infection might portend some danger in later years of life as it increases the risk of bladder cancer. In the light of the above we recommended that all children infected with urinary schistosomiasis should be screened for bacteriuria (urinary tract infection) and appropriate antibiotics concurrently administered. These children should also be followed up to monitor for later complications. The moderate prevalence of urinary schistosomiasis underscores the need for intensive health education to be carried out to sensitize the people on the risk of urinary schistosomiasis and its co-infection with bacteriuria. This could be done through community dialogue

## References

[1] Chitsulo L, Engels D, Monstesor A, Savioli L (2000). The global status of schistosomiasis and its control. Ada Trop..

[2] World Health Organization (1999). Report of WHO informal consultation on schistosomiasis control.

[3] Okoli EI, Odabido AB (1999). Urinary schistosomiasis among school children in Ibadan, an urban community in south-western Nigeria. Trop Med Int Health..

[4] World Health Organization Prevention and control of schistosomiasis and soil transmitted heminthiasis.

[5] Ozumba NA, Christiensen NO, Nwosu ABC, Nwaorgu OC (1989). Endemicity, focality and seasonality of Transmission of Human Schistosomiasis in Amagunze Village, eastern Nigeria. J Helminthology..

[6] Badawi AF, Mostafa MH, O'Conor PJ (1992). Involvement of alkylating agents in schistosome-associated bladder cancer: the possible basic mechanisms of induction. Cancer Lett..

[7] Poggensee G, Krantz I, Nordin P, Mtweve S, Ahlberg B, Mosha G, Freudenthal S (2005). A six year follow- up of children for urinary and intestinal schistosomiasis and soil transmitted heminthiasis in Northern Tanzania. Acta Trop..

[8] Latif AS (2004). Urogenital infections in the tropic. The Australasian college of Tropical Medicine. http://www.troped.org/primer/chapter.

[9] Hicks RM, Walters CL, El-Sebai I, El-Merzabani M, Gough TA (1976). Determination of nitrosamines in human urine: preliminary observations as the possible etiology for bladder cancer in association with chronic tract infections. Proc R Soc Med..

[10] Hicks RM, Ismail MM, Walters CL, Beecham PT, Rabie MT, El-Alamy MA (1982). Association of bacteriuria and urinary nitrosamine formation with Schistosoma haematobium infection in the Qalyub area of Egypt. Trans R Soc Trop Med Hyg..

[11] Hicks RM (1982). Nitrosamines as possible etiological agents in bilharzial bladder cancer. Banburry Report..

[12] Hicks RM, James C, Webbe G (1980). Effect of Schistosoma haematobium and N-butyl-N-(4-hydroxybutyl)nitrosamine on the development of urothelial neoplasia in baboon. Br J Cancer..

[13] Fincham JE, Markus MB, Adams VJ (2003). Could soil transmitted heminth infection influence the HIV/AIDS pandemic?. ActaTropica..

[14] Federal Republic of Nigeria Official Gazette (2007). Population Census 2006.

[15] Lwanga KS, Lemeshow S (1991). Sample size determination in health studies: A practical manual.

[16] Berkow R, Fletcher AJ (1987). The Merck Manual of Diagnosis and Therapy. General Medicine.

[17] Colle JG, Duguid JP, Fraser AG, Armion BJ (1989). Practical Medical Microbiology.

[18] Cowan ST, Steel JL (1975). Manual for the Identification of Medical Bacteria.

[19] Uneke CJ, Ugwuoke-Adibuah S, Nwakpu KO, Ngwu BA (2009). An Assessment of Schistosoma haematobium infection and urinary tract bacterial infection amongst school children in rural eastern Nigeria. The Internet Journal of Laboratory Medicine.

[20] Laughlin LW, Farid Z, Mansour N, Edman DC, Higashi GI (1978). Bacteriuria in urinary schistosomiasis in Egypt: a prevalence survey. AmJ Trop Med Hyg..

[21] el-Mawla NG, el-Bolkainy MN, Khaled HM (2001). Bladder cancer in Africa: update. Semin Oncol..

[22] Bedwani R, Renganathan E, El Kwhsky F (1998). Schistosomiasis and the risk of bladder cancer in Alexandria, Egypt. Br J Cancer..

[23] (1994). IARC Working Group on the evaluation of carcinogenic risks to human schistosomes, liver flukes and Helicobacter pylori.

[24] Nmorsi OP, Kwandu UN, Ebiaguanye LM (2007). Schistosoma haematobium and urinary tract pathogens co-infection in a rural community of Edo State, Nigeria. J Commun Dis..

[25] Ofoeze IE (2002). Human health and sustainable water resources development in Nigeria: Schistosomiasis in artificial lakes. Natural Resources Forum..

[26] Useh MF, Ejezie GC (1999). School-based schistosomiasis control programme: A comparative study on the prevalence and intensity of urinary schistosomiasis among Nigerian school-age children in and out of school. Transactions of the Royal Society of Tropical Medicine and Hygiene..

[27] Agbolade OM, Akinboye DO, Awolaja A (2004). Intestinal helminthiasis and urinary schistosomiasis in some villages of Ijebu North, Ogun State, Nigeria. Afr J Biotechnol..

[28] Ugbomoiko US, Ofoezie IE, Okoye LC, Heukelbach J (2010). Factors associated with urinary schistosomiasis in two peri-urban communities in south-western Nigeria. Annals of Tropical Medicine & Parasitology..

[29] Morenike OA, Idowu BA (2011). Studies on the prevalence of urinary schistosomiasis in Ogun State, south western Nigeria. West Afr J Med..

[30] Nmorsi OPG, Egwunyenga OA, Okolo OE (2001). Schistosoma haematobium infections in two rural communities in Edo State, Nigeria, SouthEast Asia. J Trop Med Public Health..

[31] Nmorsi OP, Kwandu UN, Ebiaguanye LM (2007). Schistosoma haematobium and urinary tract pathogens co-infection in a rural community of Edo State, Nigeria. J Commun Dis..

[32] Atupelep Kapito Tembo, Victor Mwapara, Steven R Meshnick (2009). Prevalence, distribution and risk factors for Schistosoma haematobium infection among school children in Blantyre, Malawi. PLoS Negl Trop Dis..

[33] Ross AG, Bartley PB, Sleigh AC (2002). Schistosomiasis. N Eng J Med..

[34] Montressor A (1998). Guidelines for the evaluation of soil transmitted heminthiasis and schistosomiasis at community level.

[35] Bundy DA, Hall A, Medley GF, Savioli L (1992). Evaluating measures to control intestinal parasitic infections. World Health Stat Q..

